# Validation of Psychometric Tools for Assessing Fatigue, Mood, and Sleep Quality: Application in the PREVES-STOP Study

**DOI:** 10.3390/medicina61020218

**Published:** 2025-01-26

**Authors:** Giuseppe Di Lorenzo, Carlo Buonerba, Raffaele Baio, Oriana Strianese, Francesca Cappuccio, Antonio Verde, Alessia Nunzia Calabrese, Vittorino Montanaro, Federica Fortino, Antonio Tufano, Roberta Zarrella, Luigi Pucci, Matteo Ferro, Concetta Ingenito, Vittorio Riccio, Emma Costa, Giovanni Riccio, Carla Errico, Anna Buonocore, Ilaria Gallo, Gianluca Amoruso, Paolo Verze, Ferdinando Costabile, Luca Scafuri

**Affiliations:** 1 Oncology Unit, “Andrea Tortora” Hospital, ASL Salerno, 84016 Pagani, Italy; giuseppe.dilorenzo@unicamillus.org (G.D.L.); xt.strianeseo@aslsalerno.it (O.S.); cappuccio.francesca@tiscali.it (F.C.); antonioverde93@gmail.com (A.V.); alessianunziacalabrese@gmail.com (A.N.C.); xt.fortinof@aslsalerno.it (F.F.); xt.zarrellar@aslsalerno.it (R.Z.); concettaingenito@gmail.com (C.I.); ferdicostabile@gmail.com (F.C.); lucaluca@hotmail.it (L.S.); 2 Associazione O.R.A. ETS-Oncology Research Assistance, 84134 Salerno, Italy; 3 Department of Medicine, UniCamillus-Saint Camillus International University of Health Sciences, 00131 Rome, Italy; 4 Department of Urology, Umberto I, Nocera Inferiore, 84014 Salerno, Italy; dott.rbaio@gmail.com; 5 Urology Unit, San Leonardo Hospital, Castellammare Di Stabia, 80053 Naples, Italy; vittorino.montanaro@alice.it; 6 Department of Urology, Istituito Nazionale Tumori IRCCS Fondazione G. Pascale, 80131 Napoli, Italy; antonio.tufano91@gmail.com; 7 Department of Urology, “Antonio Cardarelli” Hospital, 80131 Naples, Italy; luigi.pucci@aocardarelli.it; 8 Department of Health Science, ASST Santi Paolo and Carlo, University of Milan, 20133 Milan, Italy; matteo.ferro@unimi.it; 9 Ospedale S. Maria della Pietà, 80026 Casoria, Italy; vittorioriccio1990@gmail.com; 10 A.O.U. Luigi Vanvitelli, Internal Medicine–Nuovo Policlinico Campus, 80138 Naples, Italy; emmy_2716@yahoo.it; 11 A.O.U. Vanvitelli, Internal Medicine–San Paolo Hospital Campus (Fuorigrotta), 20142 Naples, Italy; ricciogiovanni1992@gmail.com (G.R.); carlaerrico21@gmail.com (C.E.); 12 Primary Care Department, ASL Salerno (SA), 84016 Pagani, Italy; anna.buonocore1962@gmail.com (A.B.); ilariag.10@libero.it (I.G.); 13 Department of Medicine, Surgery and Dentistry, Scuola Medica Salernitana, University of Salerno, 84081 Baronissi, Italy; pverze@unisa.it

**Keywords:** psychometric validation, fatigue, mood, sleep quality, environmental pollution

## Abstract

*Background and Objectives*: Environmental pollution in regions like the Sarno River Basin in southern Italy significantly affects physical and psychological health. This study aimed to validate three novel psychometric tools—REST, HEAL-BDLC, and PEACE—for assessing fatigue, mood disturbances, and sleep quality in environmentally exposed populations. While correlations with heavy metal exposure will be addressed in a separate manuscript, this study focuses solely on psychometric validation. *Materials and Methods*: The PREVES-STOP Initiative recruited 88 participants aged 30–65 years from the Sarno River Basin. Participants completed psychometric questionnaires tailored to measure fatigue (REST), symptoms of depression and anxiety (HEAL-BDLC), and sleep quality (PEACE). Internal consistency, construct validity, and reliability were analyzed using Cronbach’s alpha, correlation analyses, and principal component analysis (PCA). A subgroup received a nutraceutical intervention for us to explore their responsiveness to change over a two-week period. *Results*: REST (α = 0.969), HEAL-BDLC (α = 0.962), and PEACE (α = 0.736) demonstrated strong reliability. PCA confirmed the unidimensional structure of REST and the two-component structure of HEAL-BDLC (depression and anxiety dimensions) and PEACE (insomnia and sleep quality). Correlations with established measures, such as the WHO Well-Being Index, supported construct validity. Among the intervention participants, significant improvements were observed in fatigue (−12.5 REST median score), mood (−13.0 HEAL-BDLC median score), sleep (+1.5 PEACE median score), and overall well-being (+4.0 WHO-5 median score). *Conclusions*: REST, HEAL-BDLC, and PEACE are reliable and valid instruments for assessing nuanced health outcomes in environmentally exposed populations. They hold potential for guiding public health interventions and evaluating environmental remediation impacts. These findings lay the groundwork for future studies linking psychometric outcomes with heavy metal exposure.

## 1. Introduction

Environmental pollution continues to be a critical issue worldwide, posing significant threats to both physical and mental health. According to a 2022 progress update, pollution accounts for approximately nine million deaths each year—equivalent to one in six deaths globally—making it the largest environmental risk factor for disease and premature death. Despite some progress in reducing traditional pollution sources such as household air pollution and contaminated water, modern pollution risks driven by industrialization and urbanization (e.g., ambient air pollution and lead poisoning) have increased by over 66% since 2000. These challenges are not confined to low- or middle-income regions; communities in high-income countries also experience harmful effects from toxic chemical exposures and industrial emissions, underlining the need for concerted global action [1].

Against this global backdrop, the Sarno River Basin—located in the Campania region of southern Italy—exemplifies how pollution can severely compromise local ecosystems and human health. Over the past three decades, this region has undergone extensive contamination due to industrial discharge, agricultural runoff, untreated sewage, and illegal waste disposal. As a result, dangerous pollutants, including heavy metals like lead and cadmium, have accumulated in soil, water, and air. These toxic substances not only pose acute risks to physical health—potentially contributing to cardiovascular disease, neurological damage, and other systemic conditions—but may also negatively affect mental well-being through stress, anxiety, and community disruption [2].

The geomorphological features of the Sarno River Basin further exacerbate the situation. The river’s relatively slow flow, combined with extensive agricultural development and heavy industrial presence, allows pollutants to disperse widely and settle in local sediments, impacting farmland, waterways, and surrounding habitats [1]. Taken together, these conditions highlight the urgent need for targeted preventive strategies in the Sarno River Basin and similar polluted regions worldwide.

Chronic exposure to heavy metals is associated with a range of adverse health outcomes. Lead, a potent neurotoxin, has been linked to cognitive impairments, behavioral disturbances, and renal dysfunction, even at low levels of exposure [3]. Similarly, cadmium exposure can result in renal dysfunction, skeletal damage, and an increased risk of cancers, particularly in the lungs and kidneys [4]. Emerging evidence suggests that heavy metals may also impact psychological health and subjective well-being, contributing to conditions such as anxiety, depression, fatigue, and sleep disturbances [5].

While occupational exposure to heavy metals is well documented, the effects of subliminal, non-occupational exposures—occurring at concentrations below levels typically considered hazardous—remain underexplored. These exposures often arise from contaminated water, food, and air and may not trigger immediate clinical symptoms. However, even low-level environmental exposures can exert subtle, cumulative effects on psychological functioning and subjective well-being through mechanisms such as oxidative stress, neuroinflammation, and endocrine disruption [6]. For example, cadmium’s interference with calcium- and zinc-dependent enzymes may disrupt neurotransmitter systems, potentially leading to mood disturbances and altered sleep patterns [5].

Psychometric tools are invaluable for assessing subtle health impacts, particularly those influenced by environmental factors. Instruments such as the Beck Depression Inventory (BDI) [6], the State-Trait Anxiety Inventory (STAI), the Pittsburgh Sleep Quality Index (PSQI) [7], and the Multidimensional Fatigue Inventory (MFI) [8] are widely recognized for measuring psychological and subjective health outcomes. However, these established tools are primarily designed for clinical and psychiatric contexts and may not adequately capture the nuanced neuropsychological and physiological effects induced by chronic exposure to environmental pollutants, such as heavy metals. These effects include subtle fatigue, pollutant-induced mood disturbances, and endocrine disruptions.

The newly developed REST, HEAL-BDLC, and PEACE questionnaires each address unique yet interrelated dimensions of health that are influenced by chronic pollution, while also capturing how these environmental stressors can exacerbate or complicate typical clinical presentations. The REST (Recognizing and Estimating Signs of Tiredness) questionnaire goes beyond traditional fatigue scales (e.g., MFI) by incorporating pollutant-specific somatic symptoms (e.g., bone aches and headaches) and measuring how fatigue impacts work, social, and emotional domains—factors often overlooked in existing tools—while also examining coping mechanisms such as increased caffeine intake or seeking medical help. The HEAL-BDLC (Health Evaluation for Affective Living) questionnaire, compared to well-established instruments like the Beck Depression Inventory (BDI) and State-Trait Anxiety Inventory (STAI), was designed to integrate the role of environmental stressors in triggering or worsening mood disturbances, emphasizing the overlap between emotional and physiological reactions linked to pollution (e.g., muscle tension and palpitations). Finally, the PEACE (Promoting Evaluation and Awareness of Comfort in Sleep) questionnaire expands upon widely used measures like the Pittsburgh Sleep Quality Index (PSQI) by focusing on how anxiety, early awakenings, and nocturnal disruptions may shape sleep quality in polluted environments, offering granular frequency scales that illuminate coping and adaptation processes.

A cohort study involving adults aged 30–65 years residing in the Sarno River Basin was conducted to apply these tools, focusing on a population exposed to chronic environmental pollution. By including items that address the unique influences of environmental factors, the REST, HEAL-BDLC, and PEACE questionnaires offer a more targeted approach to assessing health outcomes in contaminated environments. If validated, these tools could fill a critical gap in psychometric assessments, offering valuable insights into the intersection of chronic toxic exposures with mood, anxiety, fatigue, and sleep quality.

The PREVES-STOP findings will be disseminated through a two-step publication plan. The primary objective of this first article is to validate the psychometric properties of the REST, HEAL-BDLC, and PEACE questionnaires, focusing on internal consistency, construct validity, and reliability. The second article, which will be published separately, will examine the correlations between blood levels of lead and cadmium and the psychometric outcomes, providing critical insights into how low-level environmental exposures influence mental health and subjective well-being. This ultimate goal of the PREVES-STOP study is to contribute to public health strategies for mitigating the risks associated with environmental contamination while enhancing the quality of life for affected populations.

## 2. Materials and Methods

### 2.1. The PREVES-STOP Initiative

The PREVES-STOP Initiative, conducted between November and December 2024, was a targeted community health program aimed at enhancing the well-being of adults aged 30 to 65 years living in the Sarno River Basin. This region, recognized as one of the most polluted in Europe due to industrial waste, agricultural runoff, and contamination from heavy metals such as lead and cadmium, faces significant environmental and public health challenges. The initiative sought to address these issues by combining preventive healthcare with community engagement and educational outreach.

Organized by Associazione O.R.A. ETS in partnership with the Porpora Laboratory, the program delivered accessible healthcare services outside traditional clinical settings. Through a series of sessions, it integrated clinical assessments, psychosocial evaluations, and optional heavy metal testing to address the specific health needs of this vulnerable population.

The program consisted of three sessions conducted during November and December 2024. Two sessions were tailored to individuals experiencing chronic fatigue, while the third session was open to the general population. These sessions took place in private, community-based settings to ensure accessibility and encourage participation. Each session accommodated up to 30 participants, all of whom provided informed consent prior to their involvement.

Participants were initially invited to complete an online pre-visit questionnaire designed to gather data on their medical history, lifestyle habits, and psychosocial health. The instruments were carefully curated by experts to address key health domains, including fatigue, anxiety, and sleep quality, and were tailored to the unique needs of the Sarno River Basin population. Physical activity was assessed using a five-point ordinal scale with the following options: “Never” (0), “Less than once a week” (1), “1–2 times per week” (2), “3–4 times per week” (3), and “Every day” (4). Alcohol consumption was evaluated based on the average number of alcoholic units consumed per day, with the following categories: “Four or more units” (0), “Three units” (1), “Two units” (2), “One unit” (3), and “None” (4). An alcoholic unit was defined as approximately a 125 mL glass of wine (12% vol.), a 330 mL can of beer (4.5% vol.), or a 40 mL shot of spirits (40% vol.).

During the initial consultation, participants were greeted by trained staff and a medical doctor, who outlined the program objectives and addressed any questions. Baseline health metrics, including vital signs and blood samples, were collected to assess routine parameters such as glucose, cholesterol, liver function, and kidney health. Optional testing for heavy metals like lead and cadmium was also offered, reflecting the environmental exposure risks in the area.

For participants attending the chronic-fatigue-focused sessions, specific attention was given to their fatigue symptoms. The physician reviewed their questionnaire responses and provided tailored recommendations. Participants were offered a nutraceutical supplement, contingent upon the physician’s favorable assessment during their individual consultations. This supplement, formulated as a tonic adaptogen, was designed to alleviate fatigue and mental stress. It was administered at a dose of two tablets twice daily, providing standardized quantities of Eleutherococcus senticosus (400 mg), Rhodiola rosea (400 mg), magnesium (300 mg), and flavonoids (1 g per day in equal parts: rutin, cacao flavonols, hesperidin, quercetin, and naringin). The supplement, which is commercially available as Previflanplus^®^ Energy (WaisPharma, Milan, Italy, hereafter referred to as the Product), was procured independently by Associazione O.R.A., with no funding or other contractual relationships with the manufacturer. It was provided to participants at no cost, along with detailed guidance on its use, potential benefits, and mechanisms of action.

The adaptogenic components of the supplement are supported by scientific evidence for their ability to alleviate fatigue, improve cognitive function, and enhance sleep quality. Eleutherococcus senticosus, commonly known as Siberian ginseng, is recognized for its adaptogenic properties, which improve mental performance and resilience to stress-induced fatigue. Clinical studies [7] support its efficacy in enhancing endurance and reducing fatigue. Rhodiola rosea, another well-documented adaptogen, has demonstrated significant benefits in mitigating mental fatigue, improving concentration, and enhancing sleep quality, particularly in stressful conditions. Studies [7,8,9] also highlight that a combination of Rhodiola rosea and Nelumbo nucifera extracts improved sleep efficiency and overall well-being, with reductions in insomnia severity and improvements in sleep quality indices.

Magnesium, an essential mineral integral to energy metabolism and neuromuscular function, has been shown to reduce fatigue and improve cognitive performance, especially in individuals with suboptimal magnesium levels. A randomized controlled trial by Reno et al. [10] found that magnesium supplementation significantly reduced muscle soreness and enhanced recovery, highlighting its role in alleviating fatigue. The inclusion of flavonoids such as quercetin and hesperidin added antioxidant and anti-inflammatory benefits, counteracting oxidative stress—a common factor in chronic fatigue—and supporting overall cognitive health. Together, these components provide a comprehensive therapeutic approach to addressing fatigue through physical, mental, and metabolic resilience.

Two weeks after the initial visit, participants returned for a follow-up session. Updated online questionnaires, completed two or three days before the follow-up, served as a basis for comparing changes in fatigue, anxiety, and sleep quality. Blood test results were reviewed, and participants received personalized recommendations based on their individual health profiles. These recommendations emphasized adherence to the Mediterranean diet, regular physical activity, stress reduction techniques, and smoking cessation. The continued use of the nutraceutical supplement was reassessed to determine its ongoing relevance and effectiveness. Participants were also educated about preventive health measures, including age-appropriate cancer screenings, to promote sustained health improvements.

### 2.2. Development of the REST, HEAL-BDLC, and PEACE Questionnaires

The REST, HEAL-BDLC, and PEACE questionnaires were developed using a theory-driven, region-specific approach to address the unique health challenges faced by populations chronically exposed to heavy metals, particularly lead and cadmium, in the Sarno River Basin. A preliminary literature review highlighted the need to capture subtle but impactful symptoms—such as chronic fatigue, mood disturbances, and fragmented sleep—that, while often below clinical thresholds, significantly affect quality of life. Existing validated tools, such as the Beck Depression Inventory (BDI), Pittsburgh Sleep Quality Index (PSQI), and State-Trait Anxiety Inventory (STAI), provided a foundational framework. However, extensive consultations with local clinicians and environmental health experts revealed critical gaps. These gaps included the frequent overlap between physical and psychological symptoms and the specific presentation of regionally prevalent conditions, such as bone aches, muscle pain, and insomnia. Guided by evidence linking low-level heavy metal exposure to oxidative stress, neuroinflammation, and endocrine disruption, the questionnaires were tailored to capture region-specific health manifestations. For instance, the REST survey included items specific to bone pain, while the HEAL-BDLC questionnaire combined depressive and anxiety symptoms to reflect the intertwined nature of mental health impacts in this context. To enhance content validity, draft versions of the questionnaires were pilot-tested with local residents. The feedback emphasized the importance of addressing daily functional impairments, sociocultural coping practices (such as high coffee consumption), and pervasive concerns about health risks. This iterative refinement process, informed by expert panel reviews and factor-analytic insights, resulted in three concise and user-friendly tools. These instruments aim to provide a robust framework for quantifying subtle, environmentally linked health outcomes and informing targeted public health interventions, aligning with the primary objectives of this investigation.

### 2.3. Study Design

This retrospective observational study analyzed data from the PREVES-STOP 2024 initiative, targeting adults aged 30–65 years residing in the Sarno River Basin. Participants were eligible if they met the specified age range, reported no significant ongoing health conditions requiring active treatment, completed at least the initial questionnaire, resided in one of the municipalities of the Sarno River Basin, and provided explicit consent for the anonymized use of their data for scientific purposes. These data were obtained from a secure and anonymized database that included demographic variables such as age and sex, anthropometric measurements (e.g., weight and height), vital signs (e.g., blood pressure, oxygen saturation, and heart rate), and lifestyle factors such as smoking habits, alcohol consumption, dietary adherence to the Mediterranean diet, and self-reported medical history, including hypertension, diabetes, and cardiovascular diseases. Clinical data encompassed routine blood parameters, such as glucose, cholesterol, liver function markers, and kidney function indicators (e.g., eGFR), along with optional measurements of lead and cadmium levels to evaluate environmental exposure. Psychosocial and quality-of-life metrics were assessed using a combination of the WHO Well-Being Index, a validated tool for general health evaluation, and other psychometric questionnaires compiled by the investigators based on their expertise, including the REST questionnaire for fatigue, the HEAL-BDLC questionnaire for anxiety and depression symptoms, and the PEACE questionnaire for sleep quality. The primary objectives were to preliminarily validate these investigator-compiled tools for detecting and quantifying symptoms and to ensure their reliability and usability. Specific aims included evaluating the HEAL-BDLC questionnaire for assessing anxiety and depression, the PEACE questionnaire for measuring sleep quality, and the REST questionnaire for quantifying fatigue levels. Additionally, this study examined variations over a 14-day period between participants receiving the Product intervention and those not undergoing the intervention. Key outcomes included reductions in fatigue (REST scores), improvements in anxiety and depression symptoms (HEAL-BDLC scores), and enhanced overall well-being (WHO Well-Being Index scores). This study also monitored adverse events and evaluated adherence to the supplementation regimen to ensure safety, tolerability, and compliance among the intervention group.

### 2.4. Statistical Analysis

#### 2.4.1. Psychometric Validation of Questionnaires

The primary aim of this study was to validate three novel psychometric tools: the HEAL-BDLC questionnaire, designed to assess mood symptoms such as anxiety and depression; the PEACE questionnaire, a comprehensive instrument for evaluating sleep quality, including parameters like comfort, duration, and disturbances; and the REST questionnaire, focused on measuring fatigue levels (Supplementary Materials). For the primary aim, internal consistency was evaluated using Cronbach’s alpha, with coefficients ≥ 0.7 deemed acceptable. Construct validity was assessed through correlations with biomarkers and established measures such as the WHO Well-Being Index. Spearman correlation methods were employed to explore relationships between questionnaire scores and health outcomes, integrating demographic and survey data. Additional analyses examined whether gender or smoking status influenced questionnaire scores. Point-biserial correlation tests were used for binary and continuous variables, whereas chi-square tests evaluated associations between categorical measures.

Principal component analysis (PCA) was conducted on data from the HEAL-BDLC, REST, and PEACE questionnaires to evaluate their underlying structure. Preliminary assessments, including the Kaiser–Meyer–Olkin (KMO) measure and Bartlett’s test of sphericity, confirmed the data’s suitability for the analysis. The principal component method was applied to extract components, and Varimax rotation was used to simplify interpretation by maximizing the variance in squared loadings across factors. Factors were retained using the Kaiser criterion (eigenvalues > 1). Additionally, a scree plot was inspected to verify the appropriateness of the number of retained factors. Items with factor loadings below 0.4 were flagged for potential removal. Cross-loading items, defined as those with high loadings (≥0.4) on more than one factor with a difference between the highest loadings of less than 0.2, were also identified for further evaluation. To address multicollinearity and ensure consistent scale interpretation, all variables were standardized (z-scores) prior to analysis. This step ensured that differences in variable scaling and response variability did not bias the results. Statistical analyses highlighted the tools’ potential utility in capturing nuanced variations in health-related measures and their responsiveness to interventions.

The secondary aim of this study was to examine variations in questionnaire scores between visit 1 and visit 2, comparing participants who used the supplement to those who did not, to provide additional confirmation of the validity of the questionnaires over time Descriptive statistics summarized continuous variables (e.g., means and standard deviations) and categorical variables (e.g., frequencies and percentages), while inferential analyses included the Wilcoxon signed-rank test or *t*-test for paired comparisons to assess changes in fatigue, sleep quality, and mood symptoms after two weeks among participants treated with the supplement compared to those who were not.

#### 2.4.2. Management of Missing Data

Given the small sample size, we anticipated minimal missing data and predefined a systematic approach to address it. All data collection forms were reviewed promptly for completeness, and omissions due to administrative errors were corrected when feasible through participant contact or supplemental data sources. For psychometric scales (REST, HEAL-BDLC, and PEACE), mean item imputation was predefined for cases where ≤20% of items on a scale were missing, preserving scale scores and sample size. If >20% of the scale items were missing, the scale score was set to missing and excluded from analyses involving that scale. Participants who failed to complete entire questionnaires were excluded from analyses involving those instruments but remained included in analyses for other available data. For longitudinal analyses, participants missing all follow-up data were excluded from pre–post comparisons, with baseline data retained for descriptive summaries. These predefined methods were designed to minimize bias while maintaining clarity and consistency in statistical analyses.

Statistical analyses were performed using R 4.4.2 (R Core Team, Vienna, Austria). Artificial intelligence (AI) tools (ChatGpt, OpenAI, San Francisco, CA, USA and Gemini, Google AI, Mountain View, CA, USA) were used to improve the style and grammar of this manuscript.

#### 2.4.3. Sample Size

To ensure a robust preliminary evaluation of the reliability of the REST, HEAL-BDLC, and PEACE questionnaires, a sample size of at least 85 respondents was chosen. Cronbach’s alpha, the primary metric for assessing internal consistency, requires an adequate sample size to produce stable and reliable estimates. Research guidelines for psychometric tool validation suggest using 5 to 10 respondents per item, which, for the 17-item HEAL-BDLC and 5-item PEACE questionnaires, corresponds to a recommended range of 85 to 170 participants. A sample size of 85 falls within this range and aligns with standards frequently employed in preliminary validation studies, which typically utilize 50 to 100 participants to balance statistical rigor with resource considerations. This sample size minimizes the standard error of alpha, allowing for sufficiently narrow confidence intervals to determine whether the tools meet the threshold of acceptable reliability (alpha > 0.7). Furthermore, a sample of 85 captures variability in the responses, ensuring the detection of any significant floor or ceiling effects and supporting accurate reliability estimates. Given the retrospective design of this study, this sample size is also resource-efficient while meeting methodological requirements.

#### 2.4.4. Ethical Considerations

Ethical approval was granted by the relevant institutional review boards (Institutional Review Board Protocol 00324, 16 December 2024). The data collected during this study were processed through a robust anonymization protocol to remove personal identifiers, ensuring that the information could not be traced back to individuals. If the data were shared with third parties, they were shared in a fully anonymized form, excluding any personal identifiers or sensitive details, thereby maintaining the highest levels of privacy and confidentiality. This study complied fully with the General Data Protection Regulation (GDPR) of the European Union, ensuring lawful, transparent data processing for specific, legitimate purposes while respecting participants’ rights to access, rectify, or request the deletion of their data. Security measures were implemented to protect the data and prevent their use beyond the stated purpose, adhering to GDPR’s principle of data minimization. Participants provided explicit consent voluntarily through a clear process that informed them of the purpose, the types of data collected, and their intended use, with the option to withdraw consent at any time without consequences.

## 3. Results

### 3.1. Study Population

A total of 143 individuals expressed interest in participating in the PREVES-STOP Initiative by registering through an online form promoted via Facebook. Participants could select one of three initiatives: two held in Pagani (SA) at a private office—one targeting fatigue and the other focused on general well-being—and a third initiative, also targeting fatigue, hosted by Fondazione Scoppa in Angri. Participants could register for only one initiative and, upon completing the registration process, were individually invited to their chosen initiative, with admission confirmed after meeting the eligibility criteria within three days of registration. During the two fatigue-focused initiatives, 61 individuals completed the required self-evaluation questionnaire and were admitted, of whom 52 attended and underwent further assessments. For the general well-being initiative, 27 individuals completed the requisite survey, resulting in an overall study population of 88 individuals available for the analysis of the primary endpoint. A total of 49 individuals from the fatigue-specific initiatives and 20 from the general well-being initiative completed the questionnaire before visit 2. The age distribution was broad, with the largest group aged 51–55 years (29.5%), followed by groups aged 41–45 years (18.2%) and 46–50 years (14.8%). Educational attainment varied, with 45.5% holding a high school diploma, 31.8% possessing a university degree, 20.5% completing middle school, and 2.3% having only an elementary school education. Common self-reported health conditions included high blood pressure (26.1%) and high cholesterol or triglycerides (26.1%), followed by autoimmune diseases (13.6%), with smaller percentages reporting kidney stones (9.1%), ulcers (5.7%), and gallstones (3.4%), while serious conditions like heart attack or diabetes were reported by only 1.1% of participants. Lifestyle habits also varied significantly: 26.6% engaged in physical activity less than once per week, 13.3% exercised 1–2 times weekly, 10.5% exercised 3–4 times weekly, and only 2.8% were physically active daily. Alcohol consumption was minimal, with 45.5% abstaining, 9.8% consuming one unit daily, and 3.5% consuming more than two units daily. Psychometric assessments revealed moderate fatigue levels, with an average REST score of 23.2 (range: 2–52), a mean PEACE sleep quality score of 6.5 (range: 0–17), and HEAL-BDLC mood symptom scores averaging 26.6 (range: 0–66) (Table 1 and Table 2). Of the 52 individuals assessed during the fatigue-specific initiatives, 44 were eligible for the intervention involving the study Product, with clinical reasons for excluding the remaining 8 individuals including lack of perceived need for supplementation (2), skepticism about the Product’s efficacy (2), hypersensitivity to magnesium (1), concurrent use of other supplements (2), and concerns about potential adverse effects (1). Among those eligible, 2 declined the supplement despite recommendation, while 42 reported regular use, with 2 experiencing mild abdominal discomfort and another 2 reporting insomnia, which prompted dosage adjustments to one tablet every 12 h without suspending use. No adverse events were reported via telephone, indicating overall tolerability of the intervention and providing valuable insights into the demographic, health, and lifestyle characteristics of this diverse study population. Overall, no missing data for psychometric questionnaires (REST, HEAL-BDLC, and PEACE) were reported in individuals completing the questionnaire. A small number of unanswered items were identified for demographic variables, such as previous cancer history or medical history, but these omissions were minimal and had a negligible impact on the overall analyses or study conclusions.

### 3.2. Reliability Analysis of REST, PEACE, and HEAL-BDLC Questionnaires

The reliability analysis of the REST, PEACE, and HEAL-BDLC questionnaires focused on their internal consistency, measured using Cronbach’s alpha. These instruments, designed to assess fatigue, sleep quality, and mood symptoms, respectively, were evaluated for their psychometric properties, with particular attention to the impact of individual items on scale reliability. The HEAL-BDLC questionnaire, aimed at assessing mood symptoms, demonstrated excellent internal consistency, with an overall Cronbach’s alpha of 0.962. Individual item analyses indicated that HEAL-BDLC Item 4 (“I had difficulty falling asleep or staying asleep”) weakened the scale slightly, as its removal raised the alpha to 0.963. Additionally, this item had the lowest item–total correlation (0.577), suggesting limited alignment with the broader construct. In contrast, items such as HEAL-BDLC Item 1 (“I felt sad or down”) and HEAL-BDLC Item 2 (“I had difficulty concentrating or making decisions”) exhibited strong item–total correlations, indicating their central role in the scale’s reliability. The REST questionnaire, designed to measure fatigue, also demonstrated excellent reliability, with an overall Cronbach’s alpha of 0.969. An analysis of individual items revealed that REST Item 10 (“How frustrated have you felt because of your fatigue?”) was less consistent with the overall construct. Its removal raised the alpha to 0.972, and it exhibited the lowest item–total correlation (0.606) among all items. In contrast, items like REST Item 1 (“How often have you felt tired, fatigued, without energy, or exhausted?”) and REST Item 5 (“How much has fatigue prevented you from performing your usual activities?”) showed strong alignment with the scale, with high item–total correlations supporting their contribution to reliability. For the PEACE questionnaire, which evaluates sleep quality, the overall Cronbach’s alpha was 0.736, indicating acceptable internal consistency. An analysis of individual items revealed variability in their contributions to the scale. Notably, the removal of PEACE Item 3 (“How often do you have difficulty falling asleep?”) slightly improved the alpha to 0.750, suggesting weaker alignment with the overall construct. Conversely, removing PEACE Item 5 (“How often do you wake up too early and struggle to fall back asleep?”) reduced the alpha to 0.632, highlighting its strong contribution to the scale’s reliability. Similarly, PEACE Item 2 (“How would you rate the overall quality of your sleep?”) demonstrated a high item–total correlation, further underscoring its importance.

### 3.3. Correlation Analysis

This study assessed the construct validity of new health-related questionnaires by examining their correlations with established health measures and with each other, with *p*-values below 0.05 deemed significant. The REST survey, which measures fatigue, correlated negatively with WHO Wellness (r = −0.467, *p* < 0.001). It also showed a strong positive correlation with HEAL-BDLC (r = 0.707, *p* < 0.001), indicating that higher fatigue levels are linked to heightened emotional distress. Similarly, the HEAL-BDLC questionnaire focused on anxiety and depression, showed a strong positive correlation with REST (r = 0.707, *p* < 0.001). It also showed a moderate negative correlation with WHO Wellness (r = −0.452, *p* < 0.001), emphasizing its sensitivity to psychological health inversely related to overall wellness. The PEACE scale, assessing sleep quality, correlated positively with WHO Wellness (r = 0.316, *p* = 0.003) and negatively with HEAL-BDLC (r = −0.327, *p* = 0.002), highlighting its connection to better sleep, improved well-being, and reduced emotional distress (Table 3). Factors such as BMI, alcohol consumption, and physical activity levels showed no significant correlations with REST, HEAL-BDLC, or PEACE scores. The point-biserial correlation analysis between the cumulative scores of the REST, HEAL-BDLC, or PEACE scores and the binary variables of smoking status and gender also revealed no statistically significant correlation. Overall, these null findings may reflect our relatively small sample size, which could limit the power to detect subtle associations.

### 3.4. Principal Component Analysis

#### 3.4.1. HEAL-BDLC

The PCA of the HEAL-BDLC questionnaire demonstrated robust evidence supporting a two-component solution that aligns with the core constructs of affective health. Preliminary diagnostics affirmed the dataset’s appropriateness for PCA, with a Kaiser–Meyer–Olkin (KMO) measure of 0.917 indicating excellent sampling adequacy and Bartlett’s test of sphericity (χ^2^ = 1463.85, *p* < 0.001) confirming sufficient inter-item correlations. Using the principal component method with Varimax rotation, two distinct components with eigenvalues exceeding one were identified, collectively explaining 69.82% of the total variance. The first component, accounting for 38.83% of the variance, included items reflecting depressive symptoms, such as persistent sadness, hopelessness, and diminished interest in pleasurable activities, interpreted as capturing the “depression” construct. The second component, contributing 30.99% of the variance, comprised items indicative of sustained worry, restlessness, and physical tension, interpreted as capturing the “anxiety” construct. Most items displayed strong and unambiguous loadings on one of these two components, underscoring their alignment with the classical symptomatology of depression and anxiety.

#### 3.4.2. REST Questionnaire

The PCA of the REST questionnaire provided compelling evidence for a unidimensional structure, indicating that the instrument primarily measures a single underlying construct associated with fatigue. Preliminary diagnostics confirmed the data’s suitability for PCA, with a Kaiser–Meyer–Olkin (KMO) measure of 0.919 reflecting excellent sampling adequacy and Bartlett’s test of sphericity (χ^2^ = 905.17, *p* < 0.001) indicating sufficient inter-item correlations. Using the principal component method, eigenvalues were computed for all 13 items, with only one component exhibiting an eigenvalue greater than one (7.90), explaining 60.7% of the total variance. The unidimensional structure was further supported by a scree plot, which showed a steep decline in eigenvalues after the first component, followed by a clear leveling off. These results strongly support the REST questionnaire as a psychometrically robust and unidimensional tool, simplifying its interpretation and enhancing its utility in both clinical and research settings for fatigue assessment.

#### 3.4.3. PEACE Questionnaire

The PCA of the PEACE questionnaire revealed a clear two-component structure that captures two key aspects of sleep: insomnia-related symptoms and overall sleep quality. Preliminary diagnostics confirmed the data’s suitability for PCA, with a Kaiser–Meyer–Olkin (KMO) measure of 0.667 and Bartlett’s test of sphericity (χ^2^ = 141.93, *p* < 0.001) indicating sufficient inter-item correlations. The analysis identified two distinct components: Component 1, representing insomnia symptoms, included items assessing difficulty falling asleep, waking during the night, and waking too early, with component loadings of 0.82, 0.79, and 0.76, respectively; Component 2, representing sleep quality, included items assessing perceived sleep quality and total hours of sleep, with component loadings of 0.85 and 0.80, respectively. Together, these two components accounted for 71.0% of the total variance, with Component 1 explaining 51.3% and Component 2 contributing 19.7%. These findings suggest that the PEACE questionnaire effectively captures two critical aspects of sleep: insomnia-related symptoms and overall sleep quality. This two-component structure highlights its utility as a robust tool for assessing sleep disturbances in both clinical and research contexts.

### 3.5. Variation in Participant Outcomes After Two Weeks

**Figure 1:**
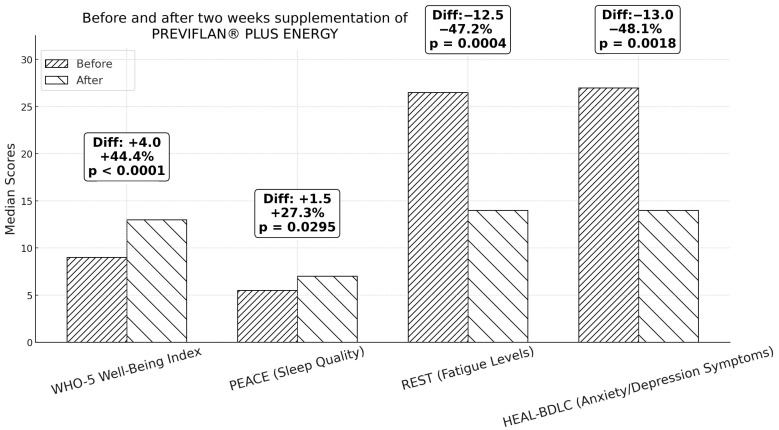
Changes in median psychometric scores before and after two weeks of PREVIFLAN^®^ PLUS ENERGY supplementation among 42 participants. For each measurement, the bar on the left represents the baseline (before intervention) values, while the bar on the right represents the post-intervention (after supplementation) values. Increases in WHO-5 Well-Being Index and PEACE (sleep quality) scores, along with decreases in REST (fatigue) and HEAL-BDLC (anxiety/depression) scores, were statistically significant (*p* < 0.05). Absolute change, percentage changes and *p*-values are shown above each pair of bars. Diff. = Difference.

Out of the original 88 participants, 69 completed the self-reported questionnaires again after two weeks. In accordance with the predefined protocol, the analysis separately assessed changes in scores between participants who underwent the intervention with the Product and those who did not. Over the two-week period, significant improvements were observed among the 42 participants who used the Product. These improvements were evident in paired data comparisons between visit 1 and visit 2. Overall well-being, as assessed by the WHO-5 Well-Being Index, showed a statistically significant increase in median scores, rising from 9.0 at visit 1 to 13.0 at visit 2. This was confirmed by the Wilcoxon signed-rank test (W = 87.0, *p* = 8.09 × 10^−6^). Similarly, REST scores, reflecting fatigue levels, demonstrated a significant decrease, with medians dropping from 26.5 at visit 1 to 14.0 at visit 2 (W = 157.0, *p* = 0.0004). Further analysis revealed significant improvements in depression/anxiety symptoms, as measured by the HEAL-BDLC questionnaire, where median scores shifted from 27.0 at visit 1 to 14.0 at visit 2 (W = 188.5, *p* = 0.0029). Sleep quality, assessed via the PEACE questionnaire, also improved significantly, with median scores increasing from 5.5 at visit 1 to 7.0 at visit 2 (W = 207.5, *p* = 0.0295). The statistical approach was guided by normality assessments performed using the Shapiro–Wilk test to determine whether parametric or non-parametric tests were appropriate. The WHO-5 scores (statistic = 0.938, *p* = 0.024), REST scores (statistic = 0.918, *p* = 0.021), and PEACE scores (statistic = 0.928, *p* = 0.040) exhibited non-normal distributions, necessitating non-parametric testing. However, the differences in the HEAL-BDLC scores followed a normal distribution (statistic = 0.985, *p* = 0.938), allowing for the application of parametric analysis. A paired *t*-test on the HEAL-BDLC scores confirmed a statistically significant improvement (t = 3.34, *p* = 0.0018), further validating the reduction in anxiety/depression symptoms (Figure 1). In contrast, among the 27 participants who repeated the questionnaires without undergoing the intervention, no significant changes were observed. The REST score had a baseline median of 18.0 and a mean difference of −0.71, with a paired *t*-test yielding a *p*-value of 0.461. The WHO-5 index had a baseline median of 11.5 and a mean difference of +0.18, with a paired *t*-test *p*-value of 0.801. The HEAL-BDLC questionnaire had a baseline median of 16.0 and a mean difference of +0.25, analyzed using the Wilcoxon test, which returned a *p*-value of 0.691. Similarly, the PEACE questionnaire had a baseline median of 5.0, a mean difference of +0.43, and a Wilcoxon test *p*-value of 0.181.

## 4. Discussion

Environmental health research has long relied on well-established instruments to connect pollutant exposure with subjective health outcomes. Interestingly, a recent Korean cross-sectional study assessed mental health via single-item questions (rather than multi-item psychometric scales) and still identified significant links between airborne metal exposure and adverse mental health outcomes, especially among individuals with asthma [11]. Conversely, a recent nationwide data linkage study employed the PSQI to examine how heavy metals in particulate matter correlated with sleep quality among adults in South Korea [12]. Although such validated scales provide broad measures of sleep disturbances, their original design does not always capture the multifaceted influence of chronic pollution stressors on fatigue, mood, and overall well-being. In response, the REST, HEAL-BDLC, and PEACE questionnaires were developed to reflect the lived experiences of populations exposed to significant pollution sources, such as those in the Sarno River Basin. Designed under the PREVES STOP Initiative, these tools offer a stable and comprehensive framework for examining fatigue, emotional distress, and sleep disturbances, while acknowledging the interplay between environmental conditions and individual health perceptions. The REST questionnaire, which adopts a unidimensional approach, simplifies interpretation for high-risk communities by generating a single fatigue score, thus reducing respondent burden relative to more segmented tools like MFI [13]. Not only does it capture subjective tiredness, but it also examines how fatigue disrupts daily activities, social interactions, future planning, and emotional well-being, offering a more comprehensive view of pollutant-induced exhaustion. In a similar vein, the HEAL-BDLC questionnaire provides a broad perspective on mood disturbances by integrating both cognitive (e.g., hopelessness and worry) and physiological (e.g., palpitations and muscle tension) components. This design offers greater insight into how environmental toxins influence both emotional and physical health. Meanwhile, the PEACE questionnaire evaluates multiple facets of sleep—namely, duration, perceived quality, and insomnia-related symptoms—to illuminate the subtle yet significant ways in which chronic pollutant exposure can undermine restorative rest. Unlike widely used measures such as the BDI [14] and the PSQI, which focus on general clinical or psychiatric contexts, these new questionnaires are expressly adapted to detect the nuanced effects of environmental contaminants on mental health and subjective well-being. For example, while the BDI is a robust tool for diagnosing depression, it does not address subtle neuropsychological manifestations of pollutant exposure—such as fatigue or neuroinflammation-driven mood changes—nor does the PSQI account for the unique challenges of low-level toxicity (e.g., cadmium’s disruption of endocrine balance and sleep). By contrast, REST broadens fatigue assessment beyond general tiredness to include pollutant-related somatic complaints (bone aches and frequent headaches) and coping behaviors (caffeine intake), domains that the MFI largely overlooks. HEAL-BDLC, by encompassing both psychological and bodily symptoms, surpasses the narrower scope of the BDI or STAI and may prove more sensitive in populations with low-level toxin exposure. Lastly, PEACE emphasizes insomnia triggers (night wakings and early awakenings) rather than relying solely on the PSQI’s global metrics, potentially offering finer-grained insights into how contaminants specifically disrupt sleep. By situating these innovative psychometric tools within the environmental and sociocultural realities of the Sarno River Basin, researchers can assess their effectiveness in capturing the health needs of a population under intense ecological pressure, where heavy metals pose risks not only to physical health but also to mental and neurocognitive well-being, especially among vulnerable groups such as children and individuals with pre-existing conditions.

This work lays the groundwork for investigating the links between psychometric findings and subliminal exposure to heavy metals, particularly lead and cadmium, which will be detailed in a forthcoming paper. In fact, chronic low-level lead exposure exacerbates oxidative stress by generating reactive oxygen species (ROS) and depleting cellular antioxidants, leading to cellular damage that manifests in neurobehavioral disorders and mood disturbances [15]. Similarly, cadmium intensifies oxidative stress while depleting essential nutrients like vitamin D, resulting in both physical and cognitive impairments [16]. Neurotoxicity and cognitive effects associated with lead exposure are particularly troubling. Research has shown that lead negatively affects children’s intelligence scores and exacerbates ADHD symptoms [17]. Among adults, prolonged lead exposure disrupts neurotransmitter systems—particularly those involving norepinephrine and acetylcholine—causing cognitive decline and mood instability [18]. Cadmium, on the other hand, impairs mitochondrial function and neurotransmitter balance, increasing the risk of neurodegenerative conditions and cognitive impairments [19]. Behavioral and developmental issues further highlight the gravity of these toxic exposures. Low-level lead exposure is strongly associated with developmental and behavioral dysfunction in children, underscoring its profound implications for mental health and socio-emotional development [20]. Although cadmium’s behavioral impacts are less statistically significant, its cumulative neurotoxic effects heighten the risk of developmental delays and neurocognitive challenges.

The REST questionnaire demonstrated exceptional reliability, with a Cronbach’s alpha of 0.969, surpassing established instruments like the Multidimensional Fatigue Inventory (MFI), which typically achieves values around 0.84 [21]. The higher alpha suggests robust reliability for measuring fatigue symptoms, although potential item redundancy should be explored. Similarly, the HEAL-BDLC questionnaire achieved a Cronbach’s alpha of 0.962, comparable to widely used tools like the BDI-II, which reports alpha values between 0.91 and 0.93 [22]. These findings suggest that the HEAL-BDLC may be a reliable tool for assessing mood symptoms, particularly in the general population dwelling in areas of high environmental pressure. The PEACE questionnaire, with a Cronbach’s alpha of 0.736, displayed acceptable internal consistency similar to the Pittsburgh Sleep Quality Index (PSQI), which typically reports values around 0.70 [23]. The bifactorial structure of the PEACE questionnaire, which captures both insomnia-related symptoms and overall sleep quality, aligns with its intended purpose but highlights areas for refinement, such as addressing weaker contributions from specific items like PEACE Item 3.The construct validity of these tools was supported by significant correlations with established measures. Fatigue, as measured by the REST questionnaire, showed strong positive correlations with mood disturbances assessed by the HEAL-BDLC, underscoring the interplay between physical and psychological symptoms. Similarly, the PEACE questionnaire’s positive correlation with the WHO Well-Being Index and negative correlation with HEAL-BDLC scores affirmed its capacity to capture the relationship between sleep quality and overall mental health. These findings align with previous research validating instruments like the Fatigue Impact Scale (Cronbach’s alpha 0.966) [24] and the Multidimensional Fatigue Symptom Inventory-Short Form (Cronbach’s alpha 0.94–0.97) [25], emphasizing the reliability and relevance of the newly developed tools.

This study also observed significant changes in psychometric scores among participants who received a short-term nutraceutical intervention, providing preliminary evidence of the tools’ responsiveness to change. Significant improvements were noted in fatigue (REST scores), mood symptoms (HEAL-BDLC scores), and sleep quality (PEACE scores) after two weeks. These changes were accompanied by improvements in overall well-being, as assessed by the WHO Well-Being Index. While these findings suggest potential applications of the tools in evaluating intervention outcomes, the absence of a blinded control group limits definitive conclusions about the intervention’s efficacy.

Several limitations of this study should be acknowledged. The short two-week follow-up period restricts assessments of the long-term reliability and responsiveness of the tools. Additionally, the reliance on self-reported data introduces potential biases, and the retrospective design may limit generalizability. Finally, our non-blinded approach, although pragmatic, limits causal inferences regarding the nutraceutical intervention’s specific efficacy. Future randomized, controlled trials with extended follow-up and cross-cultural samples could validate these instruments’ applicability in diverse contexts, enabling longitudinal monitoring of how psychometric scores evolve alongside remediation initiatives or changing contamination levels. Future studies should also refine items with weaker contributions, such as REST Item 10 and PEACE Item 3. Despite these limitations, this study represents a significant advancement in psychometric assessment for populations exposed to environmental stressors. The validated tools provide a scalable and reliable method for capturing fatigue, mood, and sleep-related outcomes, addressing critical gaps in existing health evaluations. From a policy perspective, deploying REST, HEAL-BDLC, and PEACE in routine community health checks may help identify individuals at risk of serious environmental health consequences. Such data may guide resource allocation for remediation efforts, mental health services, and targeted interventions aimed at reducing pollutant exposure. By incorporating these tools into broader research and public health strategies, it is possible to enhance the understanding and management of subtle health impacts in environmentally affected populations.

## 5. Conclusions

In conclusion, the REST, HEAL-BDLC, and PEACE questionnaires exhibit strong reliability and validity, comparable to established instruments in their respective domains. Their development and validation address a critical need for context-specific tools capable of capturing nuanced health outcomes, particularly in populations exposed to environmental risks. Looking ahead, validating these tools in larger, more diverse cohorts can solidify their applicability in various cultural and environmental contexts. Linking REST, HEAL-BDLC, and PEACE scores with specific biomarker data—such as oxidative stress markers or detailed metal quantification—could further elucidate the biological pathways of pollutant-induced health effects. Over time, these questionnaires may serve as valuable indicators of progress as remediation efforts mitigate contamination in the Sarno River Basin, ultimately guiding evidence-based policies and public health interventions.

## Figures and Tables

**Table 1:** Descriptive statistics of continuous variables measured at baseline.

Variable	Mean	95% Confidence Interval (CI):	Median	Interquartile Range
BMI	26.50	25.43, 27.57	25.95	23.51, 28.56
WHO Wellness Index	10.09	9.08, 11.10	10.0	6.75, 13.00
REST score	23.17	20.59, 25.75	22.0	13.75, 33.25
HEAL-BDLC score	26.59	22.96, 30.22	24.5	12.00, 38.25
PEACE score	6.47	3.63, 7.30	6.0	3.75, 9.25

**Table 2:** Descriptive statistics of categorical variables measured at baseline.

	Category	Count	Percentage
Gender	Female	66	75.0
	Male	22	25.0
Age	51–55	26	29.5
	41–45	16	18.1
	46–50	13	14.7
	36–40	10	11.3
	56–60	7	7.9
	66–70	6	6.8
	31–35	5	5.6
	61–65	5	5.6
Education	High School Diploma	40	45.4
	University Degree	28	31.8
	Middle School Diploma	18	20.4
	Elementary School Diploma	2	2.2
Medical History	None	41	46.5
	High Blood Pressure	23	26.1
	High Cholesterol or Triglycerides	23	26.1
	Autoimmune Diseases	12	13.6
	Kidney Stones	8	9.0
	Stomach or Duodenal Ulcer	5	5.6
	Gallstones	3	3.4
	Heart Attack	1	1.1
	No Answer	1	1.1
	Diabetes	1	1.1
History of Cancer	No Cancer	85	96.5
	Prostate Carcinoma	1	1.1
	No Answer	2	2.2

**Table 3:** Spearman correlation analysis.

Variable	BMI	WHO Wellness Score	REST Score	HEAL-BDLC Score	PEACE Score: Quality of Sleep	Alcohol Consumption	Physical Activity
BMI	1.000 (*p* < 0.001)	0.026 (*p* = 0.810)	−0.071 (*p* = 0.511)	−0.098 (*p* = 0.366)	−0.094 (*p* = 0.382)	−0.051 (*p* = 0.636)	−0.115 (*p* = 0.286)
WHO Wellness score	0.026 (*p* = 0.810)	1.000 (*p* < 0.001)	−0.467 (*p* < 0.001)	−0.452 (*p* < 0.001)	0.316 (*p* = 0.003)	0.052 (*p* = 0.630)	0.044 (*p* = 0.681)
REST score	−0.071 (*p* = 0.511)	−0.467 (*p* < 0.001)	1.000 (*p* < 0.001)	0.707 (*p* < 0.001)	−0.175 (*p* = 0.104)	0.067 (*p* = 0.536)	0.101 (*p* = 0.347)
HEAL-BDLC score	−0.098 (*p* = 0.366)	−0.452 (*p* < 0.001)	0.707 (*p* < 0.001)	1.000 (*p* < 0.001)	−0.327 (*p* = 0.002)	0.056 (*p* = 0.606)	0.081 (*p* = 0.454)
PEACE score: quality of sleep	−0.094 (*p* = 0.382)	0.316 (*p* = 0.003)	−0.175 (*p* = 0.104)	−0.327 (*p* = 0.002)	1.000 (*p* < 0.001)	0.162 (*p* = 0.132)	−0.088 (*p* = 0.413)
Alcohol consumption	−0.051 (*p* = 0.636)	0.052 (*p* = 0.630)	0.067 (*p* = 0.536)	0.056 (*p* = 0.606)	0.162 (*p* = 0.132)	1.000 (*p* < 0.001)	0.004 (*p* = 0.971)
Physical activity	−0.115 (*p* = 0.286)	0.044 (*p* = 0.681)	0.101 (*p* = 0.347)	0.081 (*p* = 0.454)	−0.088 (*p* = 0.413)	0.004 (*p* = 0.971)	1.000 (*p* < 0.001)

## Data Availability

The data supporting the findings of this study are available upon reasonable request.

## Supplementary Materials

The following supporting information can be downloaded at: https://www.mdpi.com/article/10.3390/medicina61020218/s1, REST (Fatigue), HEAL-BDCL (Anxiety/Depression), and PEACE (Quality of Sleep) Psychometric Tools.
