# Supplementary material for: Validation of Psychometric Tools for Assessing Fatigue, Mood, and Sleep Quality: Application in the PREVES-STOP Study
Source: Medicina (Kaunas). 2025 Jan 26;61(2):218. doi: 10.3390/medicina61020218 (PMC11857234; doi:10.3390/medicina61020218)
Supplement: Supplementary file 1 [file medicina-61-00218-s001.zip › medicina-3412293-supplementary.pdf]

Supplementary Materials

REST (Fatigue), HEAL-BDCL (Anxiety/Depression), and PEACE (Quality of Sleep) Psychometric Tools

The REST SURVEY: Recognizing and Estimating Signs of Tiredness
Please answer the following questions carefully. The questions refer to your experiences over the **last 30 days**.
Please keep the following scale in mind while answering:

- 0 = Not at all
- 1 = A little
- 2 = Moderately
- 3 = Very much
- 4 = Extremely

The total score is calculated by adding up the points assigned to each question.

Questions:

1. How often have you felt tired, fatigued, without energy, or exhausted?
2. How often have you experienced muscle pain?
3. How often have you suffered from headaches?
4. How often have you felt as if your bones were aching or broken?
5. How much has fatigue prevented you from performing your usual activities (work, study, household chores, etc.)?
6. How much has fatigue limited your ability to plan the activities you want to do?
7. How much has fatigue affected your work or study life?
8. How much has fatigue affected your social life?
9. How much has fatigue affected your emotional life and intimate relationships?
10. How frustrated have you felt because of your fatigue?
11. How worried have you felt because of your fatigue?
12. How often have you felt the need to consume coffee, tea, energy drinks, or supplements to combat fatigue?
13. How often have you felt the need to seek medical help because of fatigue?

HEAL-BDLC: Health Evaluation for Affective Living
Instructions:
Please complete this questionnaire carefully. The questions refer to the last 30 days.
Keep in mind the legend:
0 = Not at all
1 = A little
2 = Moderately
3 = Very much
4 = Extremely
The total score is calculated by adding up the points assigned to each question.

Questions:

1. I felt sad or down.
2. I had difficulty concentrating or making decisions.
3. I felt hopeless about the future.
4. I had difficulty falling asleep or staying asleep.
5. I felt tired or lacking in energy.
6. I had little appetite or I overate.
7. I felt worthless or a burden to others.
8. I lost interest or pleasure in activities I used to enjoy.
9. I felt slowed down in my movements or thinking.
10. I had negative thoughts about myself or the future.
11. I felt restless or agitated.
12. I had difficulty relaxing.
13. I worried excessively about various things.
14. I felt afraid for no apparent reason.
15. I had palpitations or shortness of breath.
16. I had the feeling of having a lump in my throat or stomach.
17. I had muscle tension.

PEACE: Promoting Evaluation and Awareness of Comfort in Sleep
Please answer the following questions carefully. The questions refer to your experiences over the last 30 days.
The total score is calculated by adding up the points assigned to each question.

Questions

1. **How many hours of sleep do you get on average each night?**
   - **0** = Less than 5 h
   - **1** = Between 5 and 6 h
   - **2** = Between 6 and 7 h
   - **3** = Between 7 and 8 h
   - **4** = More than 8 h
2. **How would you rate the overall quality of your sleep?**
   - **0** = Poor
   - **1** = Fair
   - **2** = Satisfactory
   - **3** = Good
   - **4** = Excellent
3. **How often do you have difficulty falling asleep?**
   - **0** = Three or more times a week
   - **1** = Once or twice a week
   - **2** = Less than once a week
   - **3** = Less than once a month
   - **4** = Never
4. **How often do you wake up during the night?**
   - **0** = Three or more times a week
   - **1** = Once or twice a week
   - **2** = Less than once a week
   - **3** = Less than once a month
   - **4** = Never

5. **How often do you wake up too early and struggle to fall back asleep?**

- ○ **0** = Three or more times a week
- ○ **1** = Once or twice a week
- ○ **2** = Less than once a week
- ○ **3** = Less than once a month
- ○ **4** = Never
